# Easy Fabrication of Thin Membranes with Through Holes. Application to Protein Patterning

**DOI:** 10.1371/journal.pone.0044261

**Published:** 2012-08-31

**Authors:** Thomas Masters, Wilfried Engl, Zhe L. Weng, Bakya Arasi, Nils Gauthier, Virgile Viasnoff

**Affiliations:** 1 MechanoBiology Institute of Singapore, Singapore, Singapore; 2 CNRS, ESPCI Paristech, Paris, France; Université d’Evry val d’Essonne, France

## Abstract

Since protein patterning on 2D surfaces has emerged as an important tool in cell biology, the development of easy patterning methods has gained importance in biology labs. In this paper we present a simple, rapid and reliable technique to fabricate thin layers of UV curable polymer with through holes. These membranes are as easy to fabricate as microcontact printing stamps and can be readily used for stencil patterning. We show how this microfabrication scheme allows highly reproducible and highly homogeneous protein patterning with micron sized resolution on surfaces as large as 10 cm^2^. Using these stencils, fragile proteins were patterned without loss of function in a fully hydrated state. We further demonstrate how intricate patterns of multiple proteins can be achieved by stacking the stencil membranes. We termed this approach microserigraphy.

## Introduction

Protein patterning on 2D surfaces has had a significant impact in biology ranging from tissue regeneration to basic cell studies [1], [2]. Local control of protein density has proven a very efficient tool to study cell adhesion dynamics [3], cell spreading [4], [5] and cell-cell interactions [4]. In addition, compelling cells to adhere on geometric patterns has emerged as a powerful tool to “normalize” cell shape and cytoskeleton organization. As an example, it has been used to study the orientation of mitotic spindles [6], and cell division [7], [8]. Further, the ability to produce large arrays of multiple protein spots allows the fabrication of protein detection chips in the same manner as DNA chips. Accordingly, a large range of patterning techniques have been developed so far involving a variety of approaches. The advantages and drawbacks of each of them have been discussed [9] previously. Among other techniques, direct protein writing using protein spotting, dip-pen lithography [10], [11], [12] or ink-jet printing [13] were implemented to produce arrays of different proteins. Locally induced chemical reactions [14], [15], [16] or protection using laser writing [17] and deep UV etching [18] have been developed as well. Last but not least, soft lithography approaches such as micro-contact printing [19], [20] and stencil patterning [21] are also often used for simple small scale patterning. Micro-contact printing has emerged as one of the preferred laboratory techniques due to its simplicity, its versatility and its low cost (with little equipment required). An elastomeric stamp (PDMS) molded on a micro-structured silicon wafer (fabricated in house or purchased) is used to transfer dried proteins from the stamp to a substrate of interest by transient contact. Though very convenient, the technique suffers both from the lack of control over the amount of transferred protein, and from the requirement of drying the protein before stamping. It may easily result in local inhomogeneities of protein density as well as in reproducibility issues. In addition, the lack of control over the stamped protein density precludes the possibility to produce protein gradients. These problems are circumvented when using stencil patterning. In this approach the substrate is covered by a stencil membrane comprising of through holes. Proteins in solution are adsorbed onto the uncovered area and 2D patterns are obtained after removal of the stencil membrane. This technique allows for constant hydration of the proteins, and control over adsorbed protein density and homogeneity. However, stencil fabrication is often non-trivial, particularly when the required features are below 10 µm. Generally, forming thin films with a high density of through holes and a good integrity raises fabrication issues. The various approaches that were taken include fabrication of thin PDMS membranes by spin coating PDMS onto a wafer [21], [22], use of Parylene film [23], [24] and fabrication of SU8 and NOA membranes [25] prepared by UV illumination with feature size above 50 µm.

In this paper we propose an alternative fabrication technique inspired by microsticker technology [26], [27]. It provides a simple and robust way to create stencils from microcontact printing stamps with no further equipment required but a UV lamp. The stencils are easy to manipulate and possess intrinsic adhesion properties that allow their use on virtually any dry substrate. Our approach thus combines the advantages of microcontact printing and stencil patterning. By stacking the stencil membranes, we then expand our method to produce intricate patterns of various proteins and of protein at various concentrations. We termed this approach microserigraphy.

## Results

### Fabrication of the Serigraphic Membrane

**Figure 1:**
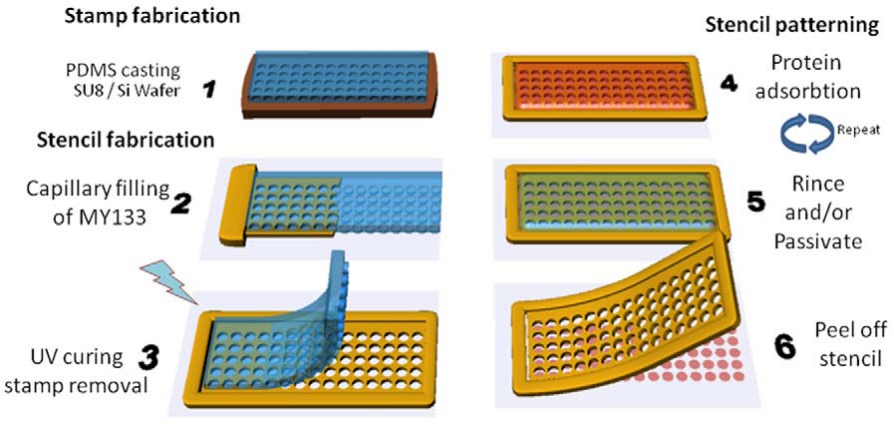
Fabrication steps for the UV curable polymer stencil membrane. A negative PDMS stamp is first fabricated using standard SU8 lithography. The stamp is then placed onto a flat substrate (e.g glass, PDMS, Petri dish). UV curable monomers are introduced by capillary flow into the gap between the stamp and the substrate. After UV curing the stamp is removed. The substrate is left for incubation with a dilute suspension of the protein of interest. Successive cycles of wash/passivation/incubation steps are then performed depending on the number of protein to be adsorbed on the patterns. After final washing the stencil is removed. Further incubation with an antifouling agent or another protein can then be performed. Notice that the proteins stay hydrated throughout the process.

Microserigraphy relies on the local adsorbtion or chemisorbtion of proteins through serigraphic stencils. Such stencils are membrane with through holes that can be stacked and deposited on any surface. The membranes are made from a UV curable polymer that allows repositionable adhesion to glass and to most plastic surfaces. The technique does not require sophisticated microfabrication processes, is readily adaptable from microcontact printing techniques and offers the advantage of stencil patterning regarding the control of coating density and large scale homogeneity. The membranes are fabricated in two steps illustrated in Figure 1. First a master mold with negative patterns is fabricated in SU8–3050 resist on a silicon wafer using standard lithography techniques. We found that a good range for the SU8 layer thickness lies between 15 to 40 µm for feature sizes ranging from 5 µm to 1 mm. It ensures both a good spatial resolution for feature development (due to a small aspect ratio) and a good final solidity of the polymer membrane. The master mold is then silanized for 2 hours under vacuum with TriChloro (1H,1H,2H,2H) perfluorooctyl silane (Aldrich). PDMS (poly dimethyl siloxane) is then repeatedly cast onto the mold and cured for 2 hours at 80°C. The PDMS stamp should be around 1 mm thick to allow enough pliability for an easy detachment from the final UV curable polymer membrane. This first step is essentially similar to fabricating a stamp for micro-contact printing.

The stencil membrane is then produced as follows. The PDMS stamp is cut such that the patterned region reaches its edge. It is then gently laid either directly on the final substrate to be coated(Glass, Petri dish, PDMS) or onto another flat substrate from which the membrane will be subsequently peeled (typically flat PDMS or plastic film). A drop of a UV curable polymer is deposited on one edge and fills the gap between the substrate and the stamp by capillarity. Among the tested UV curable monomers (NOA family, MyPoly family) the best results were obtained using MY133 DC (MyPolymer ™). Its viscosity (2500 cP) is low enough to allow smooth capillary flow. Once polymerized, it forms an elastic membrane easy to manipulate due to its Young’s modulus of 4 MPa and an elongation before breaking of 40%. It binds poorly to glass and leaves no tacky residue on the substrate after peeling when cured under water for 5 minutes with a 300 W UV lamp (Newport Model 66902). After curing, the PDMS stamp is removed. The free standing stencil membrane can be carefully peeled from the fabrication substrate. The stencil is applied to the final clean substrate by placing one edge in contact with the surface and gradually lowering it while maintaining a slight bend at the contacting region. Firmer adhesion is finally achieved by gently flattening the stencil with a thin PDMS slab.

### Single Protein Serigraphy

The principle is to use the membrane as a stencil for local adsorbtion or chemisorbtion of the proteins onto the substrate [21], [28]. The substrate with the laminated membrane is immersed in a buffer solution and degassed for 5 minutes to remove any bubbles that could remain trapped in the membrane holes. The buffer is then replaced by a dilute solution of the protein of interest at a typical concentration of 0.1 to 10 µg/ml and left for adsorbtion between 10 min to 2h depending on the desired final concentration of protein on the pattern. The substrate is washed with buffer before the membrane is carefully peeled off using tweezers in hydrated conditions. A subsequent incubation with an antifouling agent (PEG-PLL at 1 µg/ml for 1h for glass substrate, Pluronics™ at 0.2% for 1h for plastic substrate or PEG-MA at 1% for 10 min for MY133 coated substrates) can be performed to allow specific adhesion of cells to the patterns. This micro-serigraphic approach provides superior homogeneity in protein patterning since it is based on liquid adsorbtion and not on contact or dry deposition.

**Figure 2:**
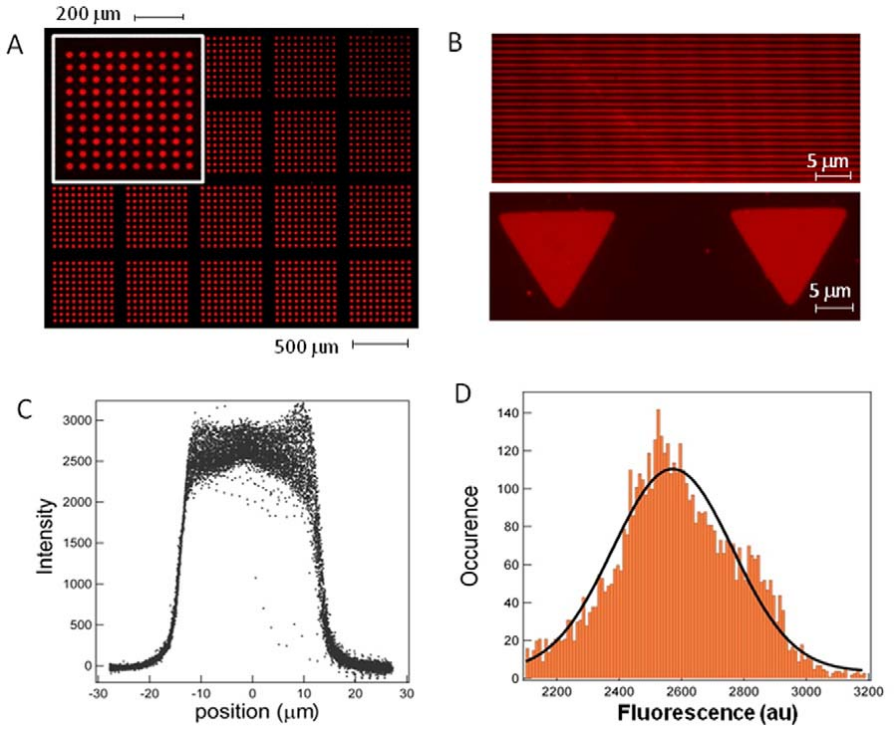
Reproducibility and accuracy tests for stencil patterning. A : Circular patterns of BSA-Alexa555 (30 µm in diameter) on a glass substrate. The total covered area is 50,000 patterns. The inset is a close-up over a subset of 100 patterns. **B**: top: 1 µm wide lines patterned on a glass slide. Bottom: 15 µm triangle with vertex of 1.5 µm radius of curvature. Micron scale precision patterning can be achieved. **C**: Overlay of 400 intensity profiles of the patterns along their diameter. **D**: Intensity distribution of the patterns. A 7% standard deviation in protein coating is measured.

As a start, we established the degree of homogeneity and reproducibility of the method using our membranes. A BSA-Alexa555 (Sigma Aldrich) solution at 10 µg/ml was incubated on a clean (nitric acid washed) glass substrate through 40 µm thick membranes with round holes of 30 µm in diameter. The typical area covered is around 1 cm^2^. The resulting patterns are illustrated in Figure 2A. The intensity profiles for each pattern are displayed in Figure 2C. The variation of intensity over a single pattern was estimated to be around 5% (std deviation). The distribution of intensity levels over the 400 patterns shown in 2A are plotted in Figure 2 C–D giving a standard deviation of 7%. These results were obtained for incubation times larger than 30 minutes. Protein depletion was sometimes noticed near the edges for short incubation times due to competing adsorbtion of the protein onto the membrane. Large stencils were then produced to cover a 10 cm Petri dish with 50,000 patterns of 30 µm each (Figure 2A and Supplementary movie S1). The homogeneity of the patterning was checked by randomly picking 400 patterns over the entire area. The distribution of their average intensity presents a standard deviation of 9%. Substrate to substrate reproducibility was also tested and was found to be around 10% (n = 10). Next we evaluated the spatial resolution protein patterns formed by this approach. Figure 2B shows examples of 1 µm wide lines patterned on a glass substrate as well as triangles of 15 µm with their vertex displaying a 1.5 µm radius of curvature much alike the PDMS stamp they originate from. Indeed we found that the limiting factor is the precision of the PDMS stamp since no shrinkage or deformation was noticed during the stencil fabrication and patterning. We conclude that the use of these MyPolymer™ stencils allow highly reproducible patterning of proteins over large surfaces with micron size resolution.

**Figure 3:**
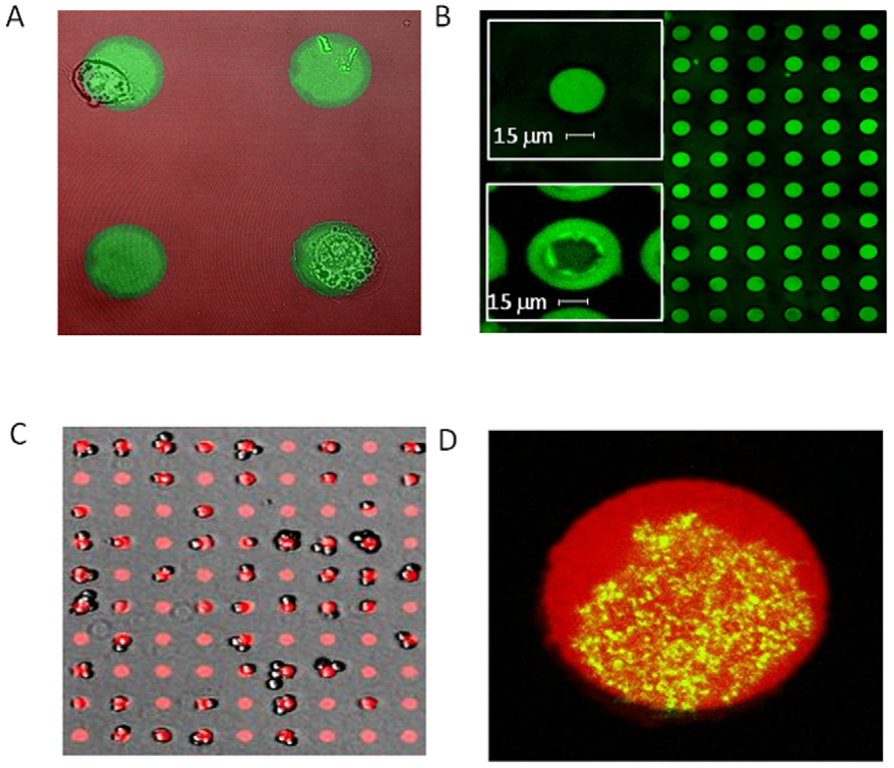
Spreading assays and supported bilayers. **A**: Spreading assay of macrophages on target antibodies. 30 µm patterns of BSA-Alexa647-mouse anti-BSA IgG and complementary coating with BSA-Alexa568. A brightfield image of macrophages spread onto the activating IgG area is overlaid. Post staining with secondary anti-mouse -Alexa 488 (green) indicates a specific coating of the anti BSA antibody on the pattern. **B**: Array of NBD-PC lipid bilayers made by stencil patterning. Top inset: close up of an individual patterned lipid bilayer. Notice the sharp boundaries with the rest of the BSA-Alexa555 passivated substrate. Bottom inset: the same pattern when the stencil is removed without BSA. The lipid bilayer radially spreads and typically dewets the glass in the centre. **C**: 10x10 arrays of E-cad patterns with adhering S180 cells. **D**: Overlay of the E-cad pattern (red stain with protein A-Alexa 647) and the endogeneous E-cadherin-GFP adhesion puncta at the glass/cell interface.

Since the stencilling process happens entirely under aqueous conditions, fragile proteins can be patterned directly without loss of function. As an example, we created two spreading assays on a glass surface patterned by 10×10 arrays of 30 µm discs. The first assay is intended to probe the immune response of macrophages to patterns of opsonin [29]. Following the protocol described in Materials and Methods, we locally patterned mouse anti-BSA antibodies onto a homogenously BSA coated surface (Figures 3A ). The specificity of the patterning was tested using fluorescently labelled antimouse secondary antibodies. Figure 3A shows the sharp patterning that we obtained. RAW macrophages were seeded onto the array and observed spreading on the antigen presenting surface. Spreading occurs exclusively on the patterns. Additionally, high resolution microscopy can be implemented on such glass substrates. Hence, the influence of the pattern size and opsonin on the dynamics and the molecular mechanism of phagocytosis can be addressed by this method.

The second assay involves patterning E-cadherin to probe the spreading of S180 murine sarcoma cells [30] cells and mimic cell-cell junction interactions. We used Recombinant Human E-Cadherin Fc Chimera (R&D systems). The patterning protocol is described in Materials and Methods. Using S180 cells stably transfected with E-cad-GFP we could image the spatial organization of the cadherin mediated adhesion patches in full or partial spreading conditions. A typical example of the E-cad distribution is given in Figure 3 C–D. Restriction of E-cad adhesion pattern resulted in a dotted structure of the adhesion sites compare to the more elongated and radial structures observed for fully spread cells.

We lastly tested the possibility to use patterned lipid bilayers [31], [32] in 15 µm patches that could be subsequently decorated with E-cad moiety. As a first step we used our stencil approach to pattern supported bilayers formed by local fusion of lipid vesicles on a nitric acid washed coverslip (see Materials and Methods). The results are shown in Figure 3B. To prevent the bilayer from spreading after stencil removal, we find it critical to peel the membrane in a buffer containing 1 µg/ml BSA. If peeled in PBS only, the membranes spread and most often rupture in their centre as shown in Figure 3B’s Inset. We found that the membrane stability exceeds one day at 4°C. Using FRAP we measured the diffusion coefficient of the lipids on the bilayer and found values ranging between 0.1 and 0.9 µm^2^/s which lies in the lower range for lipid diffusion in a supported bilayer. Further tethering of functional E-cad moiety to the lipid bilayer is currently under scrutiny.

### Multiprotein Serigraphy using Single or Multiple Serigraphic Layers

In this section we show how the stencil membrane can be used to pattern multiple proteins on the same substrate. We exemplify two cases of multiple coatings: *i-*A single protein is patterned in a background of another using a single stencil membrane, *ii-*Intertwined patterns of multiple proteins or of a single protein at various concentrations are formed in a background of a different protein using stacked serigraphic membranes.

**Figure 4:**
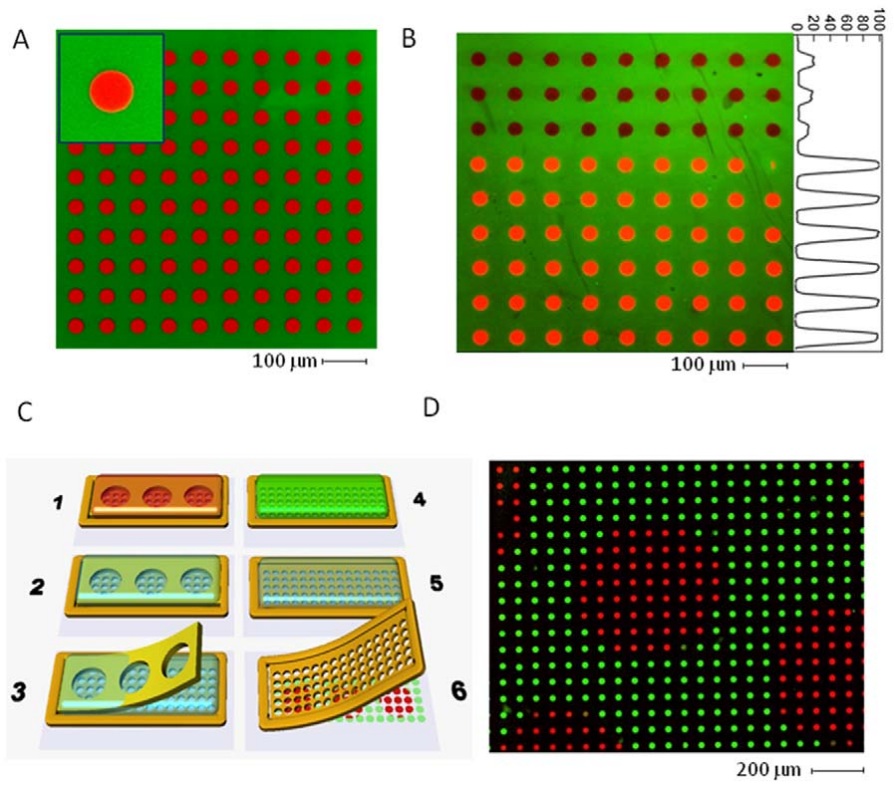
Multiprotein coating assays. **A**: BSA-Alexa555 round pattern on glass (30 µm diameter) surrounded by Fibrinogen-Alexa488. Notice the excellent complementarity of the coating of both proteins (Inset). **B**: Same protein but the top 3 rows of wells were incubated with 1 µg/ml BSA for only 20 minutes (see text for details) whereas the bottom rows were incubated with 10 µg/ml BSA to saturation. Right panel displays the average intensity of Alexa555 over a column showing that 20 minutes incubation with 1 µg/ml leads to a 20% saturation protein coating. Notice also the absence of cross contamination with the fibrinogen even in the low density zone due to the passivation step. **C**: Principles of multilayer stencilling to fabricate intertwined patterns of proteins. Stencil membrane are stacked and peeled one by one after each incubation/passivation step.1- first incubation on stacked layers,2- Rinse/Passivate, 3- Peel off the first layer,4- second incubation 5- Rinse/Passivate, 6- peel off last layer. **D**: 300 µm islands of 30 µm patterns of BSA-Alexa555 amidst patterns of BSA-Alexa488 obtained by multilayer stencilling.

To achieve multiple protein coating, the adsorbtion step for each protein is followed by an additional protection step before the membrane is lifted off. Incubation with an antifouling agent (e.g. PEG-PLL or Pluronics) prevents any further adsorption of proteins onto the pattern unless driven by specific interactions. Proteins can thus be patterned below their saturation density without cross contamination during subsequent incubation steps, or loss of function. Figure 4A displays an example of BSA-Alexa 555 patterns surrounded by Fibrinogen–Alexa488. Proteins were adsorbed at saturation (Figure 4A) and at 20% saturation (Figure 4B) on the same substrate. This demonstrates the possibility to establish discrete protein density gradients with an excellent complementarity of the patterns as shown in the inset.

The difference in BSA concentration was achieved as follows. Half of the stencil was incubated with BSA at 1 µg/ml for 20 minutes, and then passivated (with Pluronics) to prevent further protein adsorption. The entire stencil was further incubated with BSA at 10 µg/ml until saturation was reached (2 hours). A second passivation step was performed before the stencil was removed and incubated with Fibrinogen-Alexa488. Concentration (Figure 4B) profiles show that patterns with a reduced protein coating density (20% of saturation) were obtained for the first incubated area with no cross contamination from the subsequent incubation steps. Other proteins such as fibronectin were tested with equally successful results (data not shown).

The stencil membranes can also be stacked and laminated on top of each other, leading to a tight and repositionable seal. Using this property, intertwined patterns of multiple proteins can be created using a serigraphic effect. After membrane are stacked on each other, they can be peeled one by one after an incubation step for each protein of interest as shown on Figure 4C. The degasing/adsorbtion/antifouling protection cycle is repeated after every peeling step. As an example, Figure 4D shows how 600 µm wide clusters of 30 µm patterns of BSA-Alexa555 can be printed amongst the same patterns coated with BSA-Alexa488. To achieve this, we used two serigraphic membranes with respectively 30 µm and 600 µm holes. A first incubation/passivation step was performed with BSA-Alexa 555 after which the 600 µm (upper) stencil layer was removed. A second incubation/passivation step with BSA-Alexa488 was then performed and the 30 µm (lower) stencil removed.

## Discussion

Protein patterning has become a very useful tool in biology labs and patterned surfaces can now be bought off the shelf. Due to its relative simplicity the most prominent patterning technique is microcontact printing. As previously mentioned this technique involves the deposition of a dry film of protein onto an “inking” stamp that is subsequently transferred onto the working substrate. Since the emergence of soft lithography, stamp production has become an easy procedure. However, some proteins cannot be dried without loss of their function (e.g. E-cadherin). In addition the correct transfer of the protein from the stamp onto the working substrate largely depends upon the local properties of both surfaces and the even application of a gentle pressure on the stamp. These two intrinsic requirements make stamping a technique with low homogeneity of protein deposition over large surfaces or from sample to sample. Several approaches have been taken to circumvent this issue. The use of lithography methods and local chemical treatment using UV or laser writing is an alternative. However, those methods usually allow one type of protein to be patterned in a background of an anti fouling agent (in most of the cases) [21], [33]. Stencil patterning has also been described in the literature but its main difficulty, especially for small features, resides in the fabrication of a thin stencil. As mentioned in the introduction, several approaches have been taken that are usually difficult to implement in a biology lab. Our method to fabricate the stencils offers the advantage of simplicity. It can be readily adapted from the contact printing approach in a way that resembles the technique developed for microstickers [27]. Stencils can be reused several times [28] after drying, but with reduced quality of adhesion. As compared to microcontact printing, stencilling enables protein or lipid coating by hydrated adsorbtion onto a substrate. As we have shown here it leads to an excellent coating homogeneity, with control over the coating density by the incubation time and a large flexibility of combinations in the proteins involved. Stencils with micron sized features can be fabricated in less than 30 minutes with UV curable polymers, adding a single short fabrication step for superior results. Features from 1 to 300 µm were produced with thickness ranging from 5 to 40 µm.

In addition we found that the stencils can be stacked without additional steps and form efficient seals allowing multiple intertwined patterns to be produced exactly as in a serigraphic process. Hence we termed this approach microserigraphy. For the sake of coherence we presented all our proof of principle experiments with BSA coating. However, we also successfully tested fibronectin, fibrinogen and collagen without specific change in the protocol. The incubation time of the protein has only to be optimized according to the substrate and the desired coating density. Typical protein concentrations rage around 1 µg/ml and incubation time around 1 h.

Lastly our experiment on the E-cad coated surfaces shows that spreading assays can be built with proteins that require continuous hydration. This assay will be used to study the influence of the cell spread area on the organization of cadherin adhesion foci distribution. In the same spirit our spreading assay for macrophages on anti-BSA IgG patterns on a continuously coated substrate with BSA provides a clean platform where the cell is in contact with a continuous layer of the same protein with local signalling for adhesion. Creating such an assay using microcontact printing is far from straightforward due to the non specificity of antibody binding to a clean glass substrate and their sensitivity to drying.

We believe that microserigraphy can lead to an easy and more generalized used of protein patterning combining and extending the advantages of micro-contact printing and stencil patterning.

## Materials and Methods

### Protein Coating on Microwell Patterned Glass Surface

Protein A (from *Staphylococcus aureus*, Invitrogen) and Protein A conjugates (Alexa Fluor 647, Invitrogen) were diluted and mixed together in 1xPBS to a final concentration of 2.5 mg/ml and 0.5 mg/ml. 5 µl of this Protein A mixture was applied to a 0.25 cm^2^ microwell stencil on an acid-washed glass cover slip, incubated for 2 hours in the dark, and washed 3 times with PBS. EcadFc (human E-cad/Fc fusion protein, R&D Systems) was diluted in DPBS (with Ca^2+^ and Mg^2+^) to a final concentration of 100 µg/ml and 10 µl was applied to the patterned surface for 2 hours in the dark, and washed 3 times in DPBS (with Ca^2+^ and Mg^2+^). The microwell patterns were peeled off in DPBS (with Ca^2+^ and Mg^2+^) and the entire glass surface was treated with an anti-fouling reagent, 2% Pluronic F127 (Sigma-Aldrich Inc.), for 30 minutes to block cell adhesion to the unpatterned glass surface, and the glass surface was then rinsed with PBS.

### Cell Culture

S180 cells expressing full-length human E-Cadherin-GFP [30] were cultured in high glucose DMEM containing 10% FBS at 37°C in 5% CO2. At 70% confluence, the cells were detached with 1 ml of Cellstripper reagent (Non Enzymatic Cell dissociation Solution, Mediatech Inc.). Naïve contact was achieved by letting them recover in DMEM media with no FBS. A density of 8000 cell per cm^2^ was used for seeding and a 2 hour incubation time was applied to test if the S180 cells could adhere on the ProteinA-E-Cadherin patterned glass surface.

### Lipid Bilayer Patterning on the Glass Surface

The NBD-DOPC lipid vesicles were prepared according to standard protocols [34]. Fluorescently labeled bilayers contained 5% NBD-PC and 95% DOPC (Avanti Polar Lipids). Stencil membranes were prepared as previously described, and subsequently transferred to a new clean HNO_3_ treated glass surface. The patterned surface was rinsed with PBS and degassed at 1 mBar. 30 µl of NBD-DOPC lipids was added to 0.25 cm^2^ microwell patterned area. The stencil membrane was removed in 1 µg/ml BSA to block further spreading of lipid bilayers.

### Macrophage Spreading Assay

To prepare antibody patterned surfaces, the stencil coated glass surface was incubated with 1 mg/ml BSA-Alexa647 in PBS for 2 hours, washed with PBS, and incubated with 10 µg/ml Mouse anti-BSA antibody (Sigma). After removal of the stencil, the surface was incubated with 10 µg/ml BSA-Alexa568 and washed with PBS.

RAW 264.7 macrophages were maintained in 10% HI-FBS in DMEM at 37°C with 10% CO2. On the day of the experiment, cells were lightly scraped and resuspended in HEPES buffered RPMI containing 10% serum and gently rotated for 3 hours at 37°C to enable receptor recovery. Serum was removed 30 minutes prior to spreading. Experiments were performed in Ringers solution (150 mM NaCl, 5 mM KCl, 1 mM CaCl2, 1 mM MgCl2, 20 mM HEPES and 2 g/l glucose, pH 7.4) at 37°C and images taken on a Zeiss 710 LSM confocal microscope.

### Conclusion

We showed how microfabricated stencils with through holes and feature sizes ranging from 1 micron to 1 mm can be readily fabricated from UV curable polymer using PDMS stamps as secondary molds. We demonstrated how such stencils can be used to produce 2D patterns of protein on glass, Petri dishes and other polymeric substrates by local adsorbtion of proteins. We showed that this method allies the simplicity of micro-contact printing with the versatility and reproducibility of stencil printing. Patterns of 2 to 300 µm in size were produced with a protein density variation inferior to 8% over 10 cm^2^ without drying steps. Intertwined patterns of multiple proteins will allow investigation of cell spreading, polarization and target recognition in defined geometries.

## Supporting Information

Movie S1 Scan through the large scale patterning of a 10 cm^2^ Petri Dish with 50,000 round discs.
(WMV)
